# Exploring the association of dual use of the VHA and Medicare with mortality: separating the contributions of inpatient and outpatient services

**DOI:** 10.1186/1472-6963-7-70

**Published:** 2007-05-09

**Authors:** Fredric D Wolinsky, Hyonggin An, Li Liu, Thomas R Miller, Gary E Rosenthal

**Affiliations:** 1 Center for Research in the Implementation of Innovative Strategies in Practice (CRIISP), the Iowa City VA Medical Center, 601 Highway 6 West, Iowa City, IA, 52246, USA; 2 Health Management and Policy, College of Public Health, the University of Iowa, 200 Hawkins Drive, Iowa City, IA, 52242, USA; 3 Internal Medicine, Carver College of Medicine, the University of Iowa, 200 Hawkins Drive, Iowa City, IA, 52242, USA; 4 Biostatistics, College of Public Health, the University of Iowa, 200 Hawkins Drive, Iowa City, IA, 52242, USA

## Abstract

**Background:**

Older veterans may use both the Veterans Health Administration (VHA) and Medicare, but the association of dual use with health outcomes is unclear. We examined the association of indirect measures of dual use with mortality.

**Methods:**

Our secondary analysis used survey, claims, and National Death Index data from the Survey on Assets and Health Dynamics among the Oldest Old. The analytic sample included 1,521 men who were Medicare beneficiaries. Veterans were classified as dual users when their self-reported number of hospital episodes or physician visits exceeded that in their Medicare claims. Veterans reporting inpatient or outpatient visits but having no Medicare claims were classified as VHA-only users. Proportional hazards regression was used.

**Results:**

897 (59%) of the men were veterans, of whom 134 (15%) were dual users. Among dual users, 60 (45%) met the criterion based on inpatient services, 54 (40%) based on outpatient services, and 20 (15%) based on both. 766 men (50%) died. Adjusting for covariates, the independent effect of any dual use was a 38% increased mortality risk (AHR = 1.38; p = .02). Dual use based on outpatient services marginally increased mortality risk by 45% (AHR = 1.45; p = .06), and dual use based on both inpatient and outpatient services increased the risk by 98% (AHR = 1.98; p = .02).

**Conclusion:**

Indirect measures of dual use were associated with increased mortality risk. New strategies to better coordinate care, such as shared medical records, should be considered.

## Background

Millions of older veterans may use the Veterans Health Administration (VHA) system and Medicare because of their military service and age [1-6]. The outcomes of dual use may be both positive and negative [7-10]. On the one hand, dual use provides veterans with greater access to a more diverse menu of health services [4-6]. On the other hand, those services are delivered by two distinctly separate and non-communicative delivery systems, which decreases the likelihood of continuously coordinated care [3,10,11]. When continuity of care does not exist, especially for older adults with multiple chronic conditions, monitoring effectiveness decreases and the likelihood of medical errors and contraindicated and competing regimens increases [12]. It has been hypothesized that the lack of continuity of care increases the risk of hospitalization for ambulatory care sensitive conditions [12-14], and ultimately the risk of mortality [12,15,16].

Previously, we used data on 1,521 men who were self-respondents in the nationally representative Survey on Assets and Health Dynamics Among the Oldest Old (AHEAD) to examine the association between mortality and an indirect marker of dual use of Medicare and the VHA[16]. After adjusting for numerous covariates, we found that the independent effect of dual use was a 56.1% increased relative risk of mortality (AHR = 1.561; p = .009)[16]. Our measure of dual use, however, was based solely on the discordance between self-reported and claims-based inpatient (Medicare Part A) utilization. In this article we expand our indirect measure of dual use by incorporating outpatient services based on the discordance between self-reports and claims data (Medicare Part B). This overcomes a major limitation in our prior work by separating the risk of mortality for men associated with (a) dual use based just on inpatient services, from (b) dual use based solely on outpatient services, from (c) dual use based on both inpatient and outpatient services, from (d) veterans who only use the VHA, and from (e) veterans who only use Medicare (all of which are compared to the mortality risk of non-veteran men).

## Methods

### The AHEAD data set

The AHEAD study has been well described elsewhere [16-20]. We used the AHEAD because it provided a nationally representative probability sample of 1,521 men (897 veterans and 624 non-veterans) who were 70 years old or older and self respondents at baseline (1993), and whose survey data could be linked to their Medicare claims and the National Death Index (NDI) [21]. Medicare claims were available from January 1989 through December 1996. NDI data were available through December 2002. This provided up to a nine-year window, during which 766 men (50%) died, for examining the association of our indirect dual use measures with mortality. Because African Americans, Hispanics, and Floridians were over-sampled in the AHEAD, which relied on a multi-stage cluster sampling design, all analyses are weighted to adjust for the unequal probabilities of selection. When weighted, the sample of 1,521 men represents 4,297,113 noninstitutionalized men who were 70 years old or older in 1993.

### The dual use measures

Because the AHEAD is not linked to VHA claims, we constructed indirect measures of dual use that further elaborate our previous work[16]. Our approach builds on the literature addressing differences between self-reports and administrative records [22-30]. We have shown that in the AHEAD, the concordance of self-reports and Medicare claims was high for both any (vs. none; **κ **= .763) and the precise number of (**κ **= .663) hospital episodes over a 12-month window[19]. Thus, if a veteran over-reports his number of hospital episodes, he may be classified as a dual user based on inpatient services. In contrast, the concordance between self-reports and Medicare claims was low for both any (vs. none; **κ **= .248) and the precise number (**κ **= .347) of physician visits over a 12-month window[19]. Sensitivity analyses involving various bandwidth criteria, however, identified a threshold (± 3 physician visits) beyond which meaningful discordance exists[19]. Thus, if a veteran over-reports his number of physician visits by 3 or more, he may be classified as a dual user based on outpatient services. And, if a veteran over-reports his number of hospital episodes and physician visits, he would be classified as over-reporting on both.

This set of three binary indicators (dual use based only on hospital episodes, based only on physician visits, or based on both) can then be used to more granularly evaluate the different types of dual use by veterans. Or, it can be collapsed into a single, crude indicator of any dual use. Either way, three other indicator variables are included. One identifies veterans reporting inpatient or outpatient visits but having no Medicare claims; they may be classified as VHA-only users. A second identifies veterans who report inpatient or outpatient visits, but who do not over-report on either (i.e., they have Medicare claims); they may be classified as veterans who only use Medicare. The reference category (i.e., the omitted binary indicator) is non-veterans.

As detailed elsewhere[16], the classification protocols described above were operationalized using the following data. At baseline, each AHEAD man was asked whether he was hospitalized overnight during the previous 12 months, and if he had been, how many times this occurred[19]. Similarly, each AHEAD man was asked whether he had seen a doctor during the past year, and if so, how many times[19]. Using each AHEAD man's baseline interview date, corresponding data were then harvested from his Medicare claims. The numbers obtained from these self-report and claims-based sources were then used to classify veterans.

### Mortality

Vital status was obtained by linking the AHEAD to the NDI [21]. The NDI files indicate whether each AHEAD man died, and if so, provide the month and year of death through December 2002.

### Covariates

Because we hypothesized an independent effect on mortality from (a) any dual use of Medicare and the VHA, and from (b) each of the three types of dual use (inpatient only, outpatient only, or both), we included baseline socio-demographic, socio-economic, life style, disease history, functional limitations, and prior health services use covariates. The socio-demographic characteristics were age (in years), race (a set of dummy variables), and living alone (a binary indicator). The socio-economic factors included education (a set of dummy variables), household incomes less than $15 K (a binary indicator), total wealth less than or equal to $19 K (a binary indicator), and having private health insurance (a binary indicator). Life style characteristics were smoking cigarettes (a binary indicator), body mass (a set of dummy variables), and alcohol consumption and never having had a driver's license (binary indicators). Disease history was indexed by binary indicators for reporting having had arthritis, cancer, diabetes, stroke, hypertension, lung disease, heart disease, a previous hip fracture, or psychological problems. Functional limitation measures included: separate counts of activities of daily living (ADLs), instrumental ADLs (IADLs), and lower body limitations; separate binary indicators for fair or poor self-rated hearing, vision, and memory; a set of dummy variables for self-rated health; a binary indicator for the continued ability to operate a motor vehicle; and, separate sets of dummy variables for depressive symptom levels [31] and cognitive status [32]. Finally, health services use was measured by the self-reported number of hospital visits (a set of dummy variables) and the number of claims-based physician visits (excluding emergency department encounters), both during the year prior to baseline.

### Analytic approach

Because the month and year of death are known, proportional hazards models were used [33]. First, a multivariable proportional hazards model of mortality was estimated that included the binary indicator of any dual use of Medicare and the VHA by veterans, the binary indicator for veterans who only use the VHA, the binary indicator for veterans who only use Medicare, and all of the covariates identified above. Next, a second multivariable proportional hazards model was estimated that was equivalent to the first, with one exception. In this second model, the binary indicator of any dual use of Medicare and the VHA by veterans was replaced by the set of three dummy variables reflecting the subsets of dual use veterans (i.e., inpatient only, outpatient only, or both). Model development and assessment followed established guidelines [34-39]. Finally, in order to better understand why the association between dual use and mortality exists, we compared the top 15 primary diagnostic codes (ICD9-CMs) for hospital episodes for nonveterans, for veterans who were not dual users of the VHA and Medicare, and for veterans who were dual users.

### Institutional review

Because the research reported here involved the linkage of public use data files containing the AHEAD survey data with restricted data from the NDI files and Medicare claims, three layers of institutional review and approval were obtained. The first involved review and approval of the research and restricted data protection plans associated with the main NIH grant (R01 AG022913) by the AHEAD's Data Confidentiality Committee (DCC). These were approved by the AHEAD DCC on February 20, 2003 (#2003–006). The second layer of review and approval involved the University of Iowa Institutional Review Board (UI-IRB). The UI-IRB approved the original protocol on March 24, 2003, and has subsequently approved the protocol at all annual reviews (including appropriate modifications to incorporate the second NIH grant – R03 AG027741 – which specifically focused on dual use). The third layer of review and approval involved the Centers for Medicare and Medicaid Services (CMS). CMS approved the Data Use Agreement (DUA 14807) to access the Medicare claims for this research on March 3, 2005.

## Results

### Descriptive

**Figure 1:**
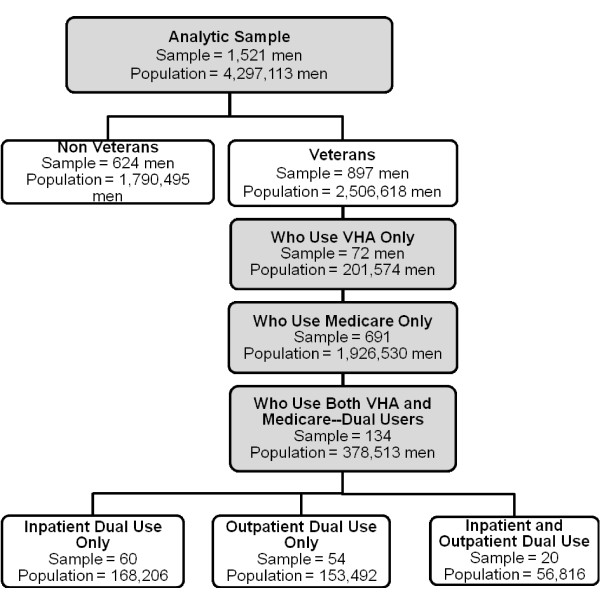
Men in the Analytic Sample and in the Population Represented by It.

Figure 1 graphically characterizes our analytic sample in terms of (a) veteran status, and among veterans in terms of (b) their use of the VHA and Medicare, and among dual users in terms of (c) the nature of their dual use. In Figure 1, each rectangle includes both the actual number of men in each group of our analytic sample, as well as the number of men in the 1993 noninstitutionalized US population 70 years old or older that they represent. As shown in the shaded top rectangle, our analytic sample consisted of 1,521 men, which represented 4,297,113 men nationally in 1993. The next level of rectangles (which are not shaded to convey that they are subsets of the rectangle above) shows that 624 men in the analytic sample were nonveterans, while 897 were veterans. In the next level of rectangles (which are again shaded to convey that they are subsets of the rectangle above), the 897 men in the analytic sample who were veterans are further classified into those who only used the VHA (72 men in the sample representing 201,574 men nationally), those who only used Medicare (691 men in the sample representing 1,926,530 men nationally), and those who were dual users of the VHA and Medicare (134 men in the sample representing 378,513 men nationally). The final level of rectangles (which are again not shaded to convey that they are subsets of the rectangle above) further classifies the dual users group into those who used both systems just for inpatient care (60 men in the sample representing 168,206 men nationally), those who used both systems just for outpatient care (54 men in the sample representing 153,492 men nationally), and those who used both systems for both inpatient and outpatient care (20 men in the sample representing 56,816 men nationally). During the nine-year follow-up 766 men (50%) died.

The three vertical panels of Table 1 show the means or percentages of the dual use markers and covariates for the 1,521 AHEAD men separately for (a) non-veterans and veterans, (b) veterans who are dual users and those who are not, and (c) veterans who are dual users by their dual use classification (inpatient, outpatient, or both). To facilitate comparisons, row (variable) entries within panels have been shaded where statistically significant differences exist. Compared to non-veterans (panel one), veterans were advantaged with regard to mortality risk in that they were younger, less likely to be minorities, had higher socioeconomic status, and better functional status. In contrast, veterans were disadvantaged relative to non-veterans because of their less healthy lifestyles, more prevalent hypertension and psychological problems, and lower numbers physician visits (claims-based). Among veterans (panel two), dual users were disadvantaged with regard to mortality status given that they were poorer, more likely to smoke, had greater disease burden, more functional limitations, and were more likely to have been hospitalized. Veterans who met both criteria had less wealth, were more likely to smoke, and were likely to have had multiple hospitalizations compared to veterans classified as dual users based only on inpatient or outpatient criteria.

**Table 1:** Analytic Sample Means or Percentages.

	**Non- Veteran**	**Veteran**	**Veteran**		**Dual Use Criteria**		
			
			**Non- Dual Use**	**Dual Use**	**By Hospitalization**	**By Physician Visits**	**By Both**
**Sociodemographic**							
Age	79.2	74.8***	74.8	74.7	75.2	74.7	73.5
Race							
White (reference group)	79.3	91.8***	92.0	91.0	94.0	87.9	90.5
Hispanic	8.8	1.8***	1.6	2.6	3.3	1.6	3.0
African American	10.6	5.7***	5.6	6.5	2.7	10.5	6.5
Other Race	1.3	0.7	0.8	0.0	0.0	0.0	0.0
Living Alone	22.1	19.4	18.9	22.0	24.0	16.1	32.1
**Socioeconomic**							
Education							
Grade School	39.0	15.8***	15.0	20.3	21.5	21.1	14.9
High School (reference group)	41.6	46.3	46.6	44.4	39.9	50.5	41.4
Some College	19.5	37.9***	38.4	35.3	38.6	28.4	43.7
Income < $15,000	38.2	18.2***	17.0	25.3*	26.8	23.1	26.5
Wealth <= $19,000	21.4	10.6***	10.0	14.0	10.9	10.8	32.0*
Private Health Insurance	75.0	87.0***	87.3	85.8	86.7	81.6	94.7
**Life Style**							
Smoker (ever)	71.3	80.9***	79.8	87.3*	91.4	78.0	100.0*
Weight							
Normal or Under Weight	47.2	40.8*	41.3	38.5	46.3	35.4	23.5
Over Weight	43.5	46.7	46.9	45.7	42.4	42.5	64.1
Obese	9.3	12.5*	11.9	15.8	11.3	22.1	12.4
Drinking							
Never drank (reference group)	52.2	36.5***	34.9	45.4*	49.4	36.8	56.9
<1 drink/day	35.4	41.2*	41.7	38.2	35.0	42.6	36.0
1–2 drinks/day	9.8	17.5***	18.5	11.9	9.8	17.7	2.4
3+ drinks/day	2.7	4.9*	5.0	4.4	5.8	2.8	4.7
Never Having a Driver's License	2.2	0.8*	0.9	0.0	0.0	0.0	0.0
**Disease History**							
Arthritis	19.1	19.4	17.9	27.6*	22.1	35.3	22.7
Cancer	12.4	15.2	14.0	21.6*	25.6	22.5	7.4
Diabetes	10.5	13.0	12.0	18.6*	11.7	22.9	27.4
Stroke	10.1	9.6	8.1	18.0***	13.4	19.0	29.3
Hypertension	37.5	44.4**	42.3	55.9**	49.3	55.9	75.5
Lung Disease	11.4	11.6	10.2	19.6**	18.4	21.2	18.6
Heart Condition	32.7	32.4	29.1	51.1***	51.6	42.3	73.5
Hip Fracture	4.1	2.1*	2.2	1.2	0.0	3.1	0.0
Psychological Problems	3.1	6.6**	5.1	14.6***	16.0	10.8	20.8
**Functional Limitations**							
ADL Counts	0.29	0.17***	0.13	0.40***	0.43	0.43	0.22
IADL Counts	0.47	0.26***	0.23	0.43**	0.46	0.41	0.40
Lower Body Limitations Count	1.07	0.78***	0.68	1.50***	1.47	1.40	1.86
Hearing – Poor or Fair	38.8	26.5***	25.2	33.8*	37.9	28.4	36.3
Vision – Poor or Fair	28.5	21.4**	19.6	31.7**	33.4	26.3	41.3
Memory – Poor or Fair	33.7	25.5***	25.2	26.9	24.6	31.0	22.4
Self-Rated Health:							
Excellent or Very Good	31.2	38.9**	42.4	19.5***	18.0	24.6	10.2
Good (reference group)	33.3	31.6	32.6	26.3	34.0	20.7	18.6
Poor or Fair	35.5	29.4*	25.0	54.2***	48.0	54.7	71.2
Ability to Drive	81.1	91.5***	92.8	84.6**	79.0	92.2	80.6
Depressive Symptoms							
None	36.8	47.7***	49.5	37.8*	37.4	41.2	30.2
1 or 2 (reference group)	39.4	35.4	35.0	37.7	32.5	43.2	38.3
3 or more	23.8	16.9***	15.5	24.5*	30.1	15.6	31.4
Cognitive Status							
Low	32.2	14.1***	13.5	17.3	22.1	14.9	9.9
Average (reference group)	30.2	35.1*	36.3	28.4	22.0	27.5	49.6
High	37.5	50.9***	50.3	54.3	55.8	57.7	40.6
**Prior Health Services Use**							
N of Self-reported Hospitalizations							
0 (reference group)	77.3	77.6	87.1	24.5***	0.0	60.4	0.0***
1	15.9	14.3	9.9	39.5***	49.3	29.3	37.7
2+	6.8	8.0	3.1	36.0***	50.7	10.2	62.3***
N of Claim-based Physician Visits	12.2	11.1*	10.6	13.9**	16.5	13.5	7.2*
**Weighted N**	634	888	754	134	60	54	20

Note: Weighted analyses adjust for the unequal probabilities of selection due either to the multi-stage cluster sampling design and/or the over-sampling. Weighted N = 1,522 due to rounding.

*p < .05 **p < .01 ***p < .001

### Proportional Hazards Regression

Table 2 contains the adjusted hazards ratios (AHRs) obtained from the two proportional hazards regression models. The results from the first model confirm our previous findings that dual use of Medicare and the VHA independently increases mortality risk[16], even though the dual use classification here could have been made on either inpatient or outpatient criteria, rather than just hospital episodes. The magnitude of the increased risk (38.3%), as well as the associations involving the covariates was also comparable. Somewhat surprising, however, was the marginally insignificant increased mortality risk associated with being a veteran who only uses the VHA, for which the AHR was nearly identical to that for dual use. We explored whether this might involve statistical confounding by adding a binary marker for self-reported service-connected disability to the first model, but the results (data not shown) were not appreciably altered. Because doing so created correlated measurement error (no comparable disability marker was available for non-veterans), we then used multivariable logistic regression [40,41] to predict VA-only use among veterans. Those results (also not shown) indicated that reporting a service-connected disability did not have an independent association, and that VA-only use was most like to occur among Hispanics, the obese, those with prior hip fractures, poor vision, and higher levels of depressive symptoms.

**Table 2:** Adjusted Hazards Ratios from the Mortality Models.

	Model 1	Model 2
**Dual Use Terms**		
Non-Veterans (reference group)	1.000	1.000
VHA Only Veterans	1.368^†^	1.379^†^
Medicare Only Veterans	1.079	1.081
Dual Use Veterans	1.383*	
Subsets of Dual Use Veterans		
Dual Use by Hospitalization Criterion		1.214
Dual Use by Physician Visit Criterion		1.449^†^
Dual Use by Both Criteria		1.979*
*Covariates*		
**Sociodemographic**		
Age	1.096***	1.096***
Race		
White (reference group)	1.000	1.000
Hispanic	0.824	0.819
African American	0.897	0.892
Other Race	1.209	1.218
Living Alone	1.060	1.051
**Socioeconomic**		
Education		
Grade School	0.807*	0.813*
High School (reference group)	1.000	1.000
Some College	0.831^†^	0.831^†^
Income < $15,000	0.923	0.929
Wealth <= $19,000	1.040	1.017
Private Health Insurance	0.963	0.961
**Life Style**		
Smoker (ever)	1.138	1.137
Weight		
Normal or under weight (reference group)	1.000	1.000
Over Weight	0.853*	0.848*
Obese	0.820	0.818
Drinking		
Never drank (reference group)	1.000	1.000
<1 drink/day	0.958	0.957
1–2 drinks/day	0.912	0.915
3+ drinks/day	1.063	1.067
Never Having a Driver's License	0.976	0.968
**Disease History**		
Arthritis	0.989	0.988
Cancer	1.202^†^	1.210^†^
Diabetes	1.340**	1.327**
Stroke	1.439**	1.428**
Hypertension	1.015	1.011
Lung Disease	1.563***	1.564***
Heart Condition	1.210*	1.200*
Hip Fracture	1.102	1.100
Psychological Problems	1.444*	1.455*
**Functional Limitations**		
ADL Count	0.940	0.942
IADL Count	1.132*	1.133*
Lower Body Limitations Count	1.062^†^	1.058
Hearing – Poor or Fair	0.945	0.941
Vision – Poor or Fair	1.170^†^	1.170^†^
Memory – Poor or Fair	0.842*	0.843*
Self-Rated Health:		
Excellent or Very Good	0.765**	0.763**
Good (reference group)	1.000	1.000
Poor or Fair	1.157	1.154
Ability to Drive	1.026	1.011
Depressive Symptoms		
None	0.964	0.968
1 or 2 (reference group)	1.000	1.000
3 or more	1.246*	1.264*
Cognitive Status		
Low	1.169	1.171
Average (reference group)	1.000	1.000
High	0.779**	0.784**
**Prior Health Services Usage**		
N of Self-Reported Hospitalizations		
0 (reference group)	1.000	1.000
1	0.922	0.922
2+	1.313*	1.328*
N of Claim-based Physician Visits	1.009**	1.009**

Note: Weighted analyses adjust for the unequal probabilities of selection due either to the multi-stage cluster sampling design and/or the over-sampling.

^† ^p < .10 *p < .05 **p < .01 ***p < .001

The results from the second model provide substantial clarification of the role of the criteria for dual use on mortality, especially given the diminished statistical power that accrues from unpacking the generic dual use marker into three specific ones. Although the only statistically significant effect involves the 98% increased mortality risk associated with being classified as a dual user on both criteria, a marginally insignificant but substantially smaller increased mortality risk (45%) was associated with dual use based only on outpatient services. In addition, although the association with veterans who only use the VA was also marginally insignificant, it also suggested increased mortality risk (38%) comparable to that of dual use based only on outpatient services. Once again, adding the service-connected disability marker did not alter this association. As expected, the effects of the covariates were equivalent to those in the first model.

### Comparing the reasons for hospitalization

Table 3 contains the top 15 primary diagnostic codes (ICD9-CMs) for hospital episodes for nonveterans, for veterans who were not dual users of the VHA and Medicare, and for veterans who were dual users. There are both similarities and differences. The greatest similarity is that for all three groups, the top 15 ICD9-CM codes account for nearly half of all hospital episodes. Furthermore, the order of the top 15 diagnostic codes is rather similar for nonveterans and for veterans who are not dual users.

**Table 3:** The 15 Most Frequent Primary ICD9-CM Codes for Hospital Episodes.

**Non Veterans**			**Veterans Who are Not Dual Users**			**Veterans Who are Dual Users**		
Code	Description	%	Code	Description	%	Code	Description	%

428	Congestive Heart Failure	6.1	414	Other Ischemic Heart Dx	5.2	780	General Symptoms	6.4
786	Resp. & hest Sx	5.6	600	Benign Prostate Hyper.	5.1	428	Congestive Heart Failure	5.4
600	Benign Prostate Hyper.	5.0	786	Resp. & hest Sx	4.9	411	Other Acute Heart Dx	4.5
486	Pneumonia	4.7	428	Congestive Heart Failure	3.9	410	Acute Myocardial Infarct	4.3
411	Other Acute Heart Dx	3.4	427	Cardiac Dysrhythmia	3.8	427	Cardiac Dysrhythmia	3.5
410	Acute Myocardial Infarct	3.2	780	General Symptoms	3.7	789	Oth. Abdomen/Pelvis Sx	3.4
780	General Symptoms	3.0	411	Other Acute Heart Dx	3.4	486	Pneumonia	3.2
414	Other Ischemic Heart Dx	2.9	410	Acute Myocardial Infarct	3.3	414	Other Ischemic Heart Dx	3.0
427	Cardiac Dysrhythmia	2.9	715	Osteoarthrosis	3.3	600	Benign Prostate Hyper.	2.9
715	Osteoarthrosis	2.7	486	Pneumonia	2.6	786	Resp. & hest Sx	2.7
578	Gastrointestinal hemor.	2.4	578	Gastrointestinal Hemor.	2.1	724	Unspecified Back Dx	2.4
436	Acute Ill-defined CVD	2.2	185	Prostate Cancer	1.9	574	Cholelithiasis	2.3
599	Other Urinary Tract Dx	2.1	599	Other Urinary Tract Dx	1.7	436	Acute Ill-defined CVD	2.3
485	Bronchopneumonia	1.8	433	Precerebrial Arter. Sten.	1.7	276	Electrolyte Imbalance	2.2
276	Electrolyte Imbalance	1.7	250	Diabetes Mellitus	1.6	715	Osteoarthrosis	2.1

Total		49.7	Total		48.1	Total		50.7

Key to Abbreviations: Resp = Respiratory; Sx = Symptoms; Dx = Diagnoses; Hyper = Hyperplasia; Infact = Infarction; CVD = Cerebrovascular Disease; Hemor = Hemorrhage; Arter = Arterial; Sten = Stenosis; Oth = Other. h

The greatest differences in Table 3 involve a somewhat different order among the 15 most frequent diagnostic codes for veterans who are dual users compared to the two other groups. Among veterans who are dual users of the VHA and Medicare, general symptoms (ICD9-CM codes 780) are much more common (6.4% vs. 3.0% or 3.7%), and other abdominal or pelvis symptoms (ICD9-CM codes 789) are the 6^th ^most frequent reason for hospitalization (3.4%), while that ICD9-CM does not even make the top 15 list of reasons for the other two groups. There are two other clear differences – the frequency of benign prostatic hyperplasia (ICD9-CM codes 600) is much less common among dual users compared to the two other groups (2.9% vs. 5.0% or 5.1%), while cholelithiasis (ICD9-CM codes 574) ranks 12^th ^among dual users (2.3%) but does not even appear on the top 15 list for the two other groups. In reviewing these data, it is important to note that these differences are not due to the use of the VHA by dual users, because all of the visits shown in the table involved hospital episodes documented by Medicare claims. What these data do indicate is that the reasons for the Medicare documented hospital episodes among dual users differ from that of nonveterans and from veterans who are not dual users of the VHA and Medicare.

## Discussion

These results contribute to the literature on the potential adverse effects of the dual use of Medicare and the VHA on mortality in two important ways. First, they extend the evidentiary base beyond a dual use marker derived solely from inpatient services[16] to include one that considers either inpatient and/or outpatient services (Model 1 in Table 2). In so doing, these results clarify that the annual period-prevalence of dual use, based on our indirect measure, was higher than previously reported (15% vs. 11%) using the same nationally representative sample of older veterans. When weighted to reflect the population of men 70 years old or older in 1993, this indicates that there are 378,513 who were dual users of the VHA and Medicare.

Second, these results unpack the generic effect of dual use by estimating the associations based on dual use classifications involving only inpatient, only outpatient, or both service types (Model 2 in Table 2). The highest increased risk involved dual use of both inpatient and outpatient services (AHR = 1.979; 95% CI = 1.068 – 3.668; which applies to 56,816 men), although a more modest and marginally insignificant effect was found for dual use that only involved outpatient services (AHR = 1.449; 95% CI = 0.64 – 1.974; which applies to 153,492 men). And it is important to note that veterans who do not use the VHA at all have mortality risks equivalent to non-veterans (the reference group; AHRs = 1.079 and 1.081 in Models 1 and 2, respectively). Although these results are not definitive, we believe that they suggest that new strategies to better coordinate care, such as shared medical records, should be considered [42-45].

What is unclear is why there was a trend (i.e., the p values for the AHRs were > .05, and thus the CIs included 1.00, although the p values were less than .10) toward increased mortality risk for veterans who only used the VHA. Based on these analyses, we suspect that this results from insufficient case-mix adjustment. We did add a marker for self-reported service-connected disability to both models, but it did not mediate the marginal effect of veterans who only use the VHA. This is likely due to two factors. First, no comparable disability measure was available for non-veterans, creating correlated measurement error. Second, selection bias not captured by the covariates was likely involved in the process of receiving care from the VHA, and given the small number of veterans using the VHA for any reason in this sample, we cannot employ propensity score methods for adjustment purposes [46-48]. Thus, further research is needed to resolve this issue. Ideally, that research would involve linkage of VHA claims to these data, which would not only provide a direct measure of dual use, but also likely increase the number of veterans identified as dual users, enhancing statistical power.

Ultimately, the big question is why is it that dual users of the VHA and Medicare have greater mortality risk? Here and previously[16] we have argued that receiving care delivered by two distinctly separate and non-communicative delivery systems decreases the likelihood of continuously coordinated care. When this happens for older adults with multiple chronic conditions, monitoring effectiveness decreases and the likelihood of medical errors and contraindicated and competing regimens increases [12,15], as does the risk of hospitalization for ambulatory care sensitive conditions [12-14], and ultimately the risk of mortality [12,15,16].

An alternative explanation is that dual users of the VHA and Medicare have substantially different illness experiences and burdens than veterans who only use Medicare, which we cannot address using the available data for case-mix adjustment. Our examination of the top 15 primary hospital diagnoses (ICD9-CM codes) in Table 3 provides some support for this. Among their Medicare claims documented hospital episodes, veterans who were dual users of the VHA and Medicare were more likely to be hospitalized for general symptoms and for abdominal and pelvic symptoms than either nonveterans or veterans who were dual users. That is, the reasons for their nonVHA hospitalizations were less well characterized, suggesting that dual users of the VHA and Medicare are facing either different or perhaps more complicated illness experiences. Unfortunately, this possibility cannot be resolved without linkage of VHA claims to the AHEAD survey data and Medicare claims.

## Conclusion

Based on our indirect measure using inpatient and/or outpatient services, male veterans who were dual users of both the VHA and Medicare had substantially greater risk of mortality (AHR = 1.38; p = .02) than their counterparts. Moreover, the number of dual users of the VHA and Medicare is not small. When weighted to reflect the population of men 70 years old or older in 1993, our results indicate that there were 378,513 dual users of the VHA and Medicare. There are two plausible explanations of why dual use increases the risk of mortality. One assumes that dual use increases the risk of uncoordinated and poorly managed care, which is especially important in the treatment and management of older adults with multiple chronic conditions. The other explanation is that the reasons for the nonVHA hospitalizations of dual users are less well characterized, suggesting that dual users of the VHA and Medicare face either different or perhaps more complicated illness experiences. Further research that links the AHEAD survey data and Medicare claims used in this analysis to VHA claims data is needed to determine which of these two explanations is most plausible.

## Competing interests

The author(s) declare that they have no competing interests.

## Authors' contributions

FDW conceived of the study, wrote both grant applications, designed the analyses, interpreted the results, and drafted and revised the manuscript. HA assisted in the design and oversight of the statistical analyses and their interpretation. LL conducted all of the statistical analyses. TRM cleaned and linked all of the data files. GER participated in the conceptualization of the grant applications and the overall study design, and provided clinical expertise. All authors read and approved the final manuscript.

## Pre-publication history

The pre-publication history for this paper can be accessed here:


